# Beneficial effects of troxerutin on metabolic disorders in non-obese model of metabolic syndrome

**DOI:** 10.1371/journal.pone.0220377

**Published:** 2019-08-12

**Authors:** Hana Malinska, Martina Hüttl, Olena Oliyarnyk, Irena Markova, Martin Poruba, Zuzana Racova, Ludmila Kazdova, Rostislav Vecera

**Affiliations:** 1 Centre for Experimental Medicine, Institute for Clinical and Experimental Medicine, Prague, Czech Republic; 2 Department of Pharmacology, Faculty of Medicine and Dentistry, Palacky University, Olomouc, Czech Republic; Max Delbruck Centrum fur Molekulare Medizin Berlin Buch, GERMANY

## Abstract

**Background:**

Troxerutin (TRX) has a beneficial effect on blood viscosity and platelet aggregation, and is currently used for the treatment of chronic varicosity. Recently, TRX can improve lipid abnormalities, glucose intolerance and oxidative stress in high-fat diet-induced metabolic disorders. In this study, we tested the effect of TRX on metabolic syndrome-associated disorders using a non-obese model of metabolic syndrome–the Hereditary Hypertriglyceridaemic rats (HHTg).

**Methods:**

Adult male HHTg rats were fed standard diet without or with TRX (150 mg/kg bwt/day for 4 weeks).

**Results:**

Compared to untreated rats, TRX supplementation in HHTg rats decreased serum glucose (p<0.05) and insulin (p<0.05). Although blood lipids were not affected, TRX decreased hepatic cholesterol concentrations (p<0.01) and reduced gene expression of HMGCR, SREBP2 and SCD1 (p<0.01), involved in cholesterol synthesis and lipid homeostasis. TRX-treated rats exhibited decreased lipoperoxidation and increased activity of antioxidant enzymes SOD and GPx (p<0.05) in the liver. In addition, TRX supplementation increased insulin sensitivity in muscles and epididymal adipose tissue (p<0.05). Elevated serum adiponectin (p<0.05) and decreased muscle triglyceride (p<0.05) helped improve insulin sensitivity. Among the beneficial effects of TRX were changes to cytochrome P450 family enzymes. Hepatic gene expression of CYP4A1, CYP4A3 and CYP5A1 (p<0.01) decreased, while there was a marked elevation in gene expression of CYP1A1 (p<0.01).

**Conclusion:**

Our results indicate that TRX improves hepatic lipid metabolism and insulin sensitivity in peripheral tissues. As well as ameliorating oxidative stress, TRX can reduce ectopic lipid deposition, affect genes involved in lipid metabolism, and influence the activity of CYP family enzymes.

## Introduction

Pharmacological interventions in individuals with metabolic syndrome and its complications can be accompanied by potential side effects. To remedy this situation, various natural solutions capable of mitigating doses of pharmacological therapy have been investigated. Previous reports have shown that bioflavonoid and polyphenol intake may be beneficial in inhibiting the onset and progression of metabolic syndrome, and in lowering the risk of developing diabetes and cardiovascular disease [1]. Apart from their expressive antioxidant and anti-inflammatory properties, some flavonoids can provide beneficial hepatoprotective effects [2], improve lipid profile [3] as well reduce blood pressure [4] and body weight [5], thus effectively attenuating the components of metabolic syndrome.

Recent studies of flavonoids have highlighted the significance of troxerutin (TRX), a trihydroxyethylated derivative of rutin. TRX is present in tea, coffee, cereal grains, and a variety of fruits and vegetables [6]. Compared with rutin, TRX can be absorbed more easily through the digestive system [7]. Clinical trials have proved that TRX has a very good safety profile and is tolerable even at high doses [8]. TRX has a beneficial effect on blood viscosity and platelet aggregation and is currently used for the treatment of chronic venous insufficiency and clinical varicosity [9].

Several other biological effects resulting from TRX administration have been reported [7]. TRX has been shown to suppress the development of lipid abnormalities and oxidative stress in the heart as well as to reduce blood glucose and hyperinsulinaemia in high-fat/high-cholesterol and fructose diet-fed rats [7] and mice [10, 11]. And since TRX has been reported to effectively reduce body weight and obesity-related metabolic parameters in high-fat diet-treated animals, its anti-obesity effects can also be inferred [12].

However, the insulin-sensitising and lipid-lowering effects of TRX have yet to be investigated, while its exact molecular mechanism is not fully understood. There have been no studies on the beneficial effect of TRX on pre-existing disorders associated with metabolic syndrome or on conditions characterised by an absence of excessive high-fat-induced obesity.

To investigate the effect of TRX treatment on metabolic syndrome abnormalities, we used a unique non-obese model of metabolic syndrome–the Hereditary Hypertriglyceridaemic rats (HHTg). Originating from the Wistar rat, this non-obese strain exhibits insulin resistance, hyperinsulinaemia, dyslipidaemia, liver steatosis, oxidative stress and low-grade chronic inflammation, and is accepted as a model of metabolic syndrome more representative of human activity [13, 14]. The aim of this study was to test the hypolipidaemic, antioxidant effect of TRX by assessing its influence on insulin resistance in peripheral tissues and on gene expression in hepatic enzymes, receptors and transcription factors.

## Materials and methods

### Animals and experimental procedure

All experiments were carried out using 5-months-old male Hereditary Hypertriglyceridaemic rats (HHTg), provided by the Institute for Clinical and Experimental Medicine, Prague, Czech Republic. The rats were maintained in a 12-h light/12-h dark cycle room at a temperature of 22–25°C and allowed free access to food and water. The animals were fed a standard laboratory diet without or with supplementation with TRX in a dose of 150 mg/kg of body weight (Cilkanol, Zentiva, Czech Republic) for 4 weeks. The standard diet consisted of 23% protein, 43% starch, 7% fat, 5% fibre, and 1% vitamin and mineral mixture (standard chow diet, Bonagro, Czech Republic). The dose of TRX was administrated in mixture of standard diet. Diet consumption was measured daily and food intake of both groups of rats did not differ. TRX has a low toxicity (rat LD50 = 27160 mg/kg) and good bioavailability (approximately 15%). Its intestinal absorption is rapid (maximum is achieved after 1.5 hour). In the body, 75% of unused TRX is metabolized in the liver, remaining 25% is eliminated by the kidney in unchanged form.

All experiments were performed in accordance with the Animal Protection Law of the Czech Republic 359/2012, which is in compliance with the European Community Council recommendations for the use of laboratory animals (86/609/ECC), and approved by the Ethics Committee of the Ministry of Health of the Czech Republic and IKEM’s Ethics Committee (Protocol Number: 28/2016). After one month of TRX treatment, the rats were sacrificed by decapitation; blood and tissues were collected and stored at -80°C. Serum and tissues homogenates were prepared as described previously [15].

### Biochemical analysis

Biochemical parameters were determined as follows: serum glucose, triacylglycerols and total cholesterol using kits from Erba Lachema (Czech Republic); insulin using an ELISA kit from Mercodia (Sveden); HDL cholesterol and NEFA using kits from Roche Diagnostics (Germany); high-molecular weight (HMW) adiponectin using an ELISA kit (MyBioSource, USA). Plasma concentrations of pro-inflammatory parameters IL-6, MCP-1 and CRP were determined by rat ELISA kits (Bio-Source International, USA; eBioscience, Austria; Alpha Diagnostics International, USA, respectively).

### Glucose utilisation in adipose tissue

Glucose utilisation in epididymal adipose tissue was analysed *ex vivo* by measuring the incorporation of ^14^C-U glucose into lipids. Distal parts of the epididymal adipose tissue were rapidly dissected and incubated for 2 hours in Krebs-Ringer bicarbonate buffer with 5 mmol/l of unlabelled glucose, 0.1 μCi/ml of ^14^C-U glucose (UVVR, Czech Republic) and 2% bovine serum albumin. The gaseous phase was set at 95% O_2_ and 5% CO_2_ in the presence or absence of insulin (250 μmol/ml) at 37°C. After incubation, adipose tissue was removed, rinsed in saline, and placed in chloroform. The pieces of tissue were dissolved after the addition of methanol (chloroform:methanol = 2:1), with lipids extracted at 4°C overnight. The next day, KH_2_PO_4_ was added before evaporating and reconstituting aliquots in scintillation liquid; radioactivity was measured by scintillation counting [16].

### Basal and insulin-stimulated glucose incorporation into glycogen in skeletal muscle

For measurement of insulin-stimulated incorporation of glucose into glycogen, soleus muscles were incubated for 2 hours in 95% O2 + 5% CO2 in Krebs-Ringer bicarbonate buffer (pH 7.4) containing 0.1 μCi/ml of ^14^C-U glucose, 5 mmol/L of unlabelled glucose and 2.5 mg/ml of bovine serum albumin (Fraction V, Sigma, Czech Republic) with or without 250 μU/ml of insulin. Glycogen was extracted, and insulin-stimulated incorporation of glucose into glycogen was determined.

In epididymal adipose tissue adrenaline-stimulated lipolysis was measured *ex vivo* according to the release of NEFA into the incubating medium.

### Parameters of oxidative stress

Concentrations of reduced (GSH) and oxidised (GSSG) forms of glutathione were determined using a HPLC kit with fluorescence detection (ChromSystems, Germany). Activity of superoxide dismutase, glutathione peroxidase, glutathione reductase, and glutathione transferase were analysed using Cayman Chemicals assay kits (MI, USA). Catalase activity was determined based on the ability of H_2_O_2_ to form a colour complex with ammonium molybdate, and detected spectrophotometrically. Concentrations of conjugated dienes were determined by extraction in media (heptane:isopropanol = 2:1) and measured spectrophotometrically in the heptane layer. TBARS levels were analysed based on the reaction with thiobarbituric acid [17].

### Gene expression profile

The relative gene expressions of hepatic enzymes, receptors and transcriptional factors were determined by real-time PCR using the TaqMan RNA-to-CT^TM^ 1-Step kit and TaqMan Gene Expression Assay (*Assays IDs*: *ABCA1-Rn00710172_m1*, *CYP1A1-Rn00487218_m1*, *CYP2E1-Rn00580624_m1*, *CYP3A-Rn00595752_m1*, *CYP4A1-Rn00598510_m1*, *CYP4A2-Rn01417066_m1*, *CYP4A3-Rn04224033_m1*, *CYP7A1-Rn00564065_m1*, *FASN-Rn00569117_m1*, *HMGCR-Rn00565598_m1*, *MCP1-Rn00580555_m1*, *NRF2-Rn00582415_m1*, *PPARA-Rn00566193_m1*, *SCD1-Rn00594894_g1*, *SREBP1-Rn01495769_m1*, *SREBP2-Rn01502638_m1;* Life Technologies, Applied Biosystems, USA) and carried out in a Vii A^TM^7 Real-Time PCR System (Applied Biosystems, USA). Relative gene expressions were determined after normalisation against β-actin (*ACTB-Rn 01412977_g1*) as an internal reference and calculated using the 2^-ΔΔCt^ method.

#### Statistical analysis

All data are expressed as mean ± SEM. Before beginning the study, χ^2^ test was used to examine qualitative variables. Statistical evaluation of data was performed using the unpaired Student’s t-test, with categorical variables analysed using Fisher’s exact test. Statistical significance was defined as p<0.05.

## Results

**Fig 1:**
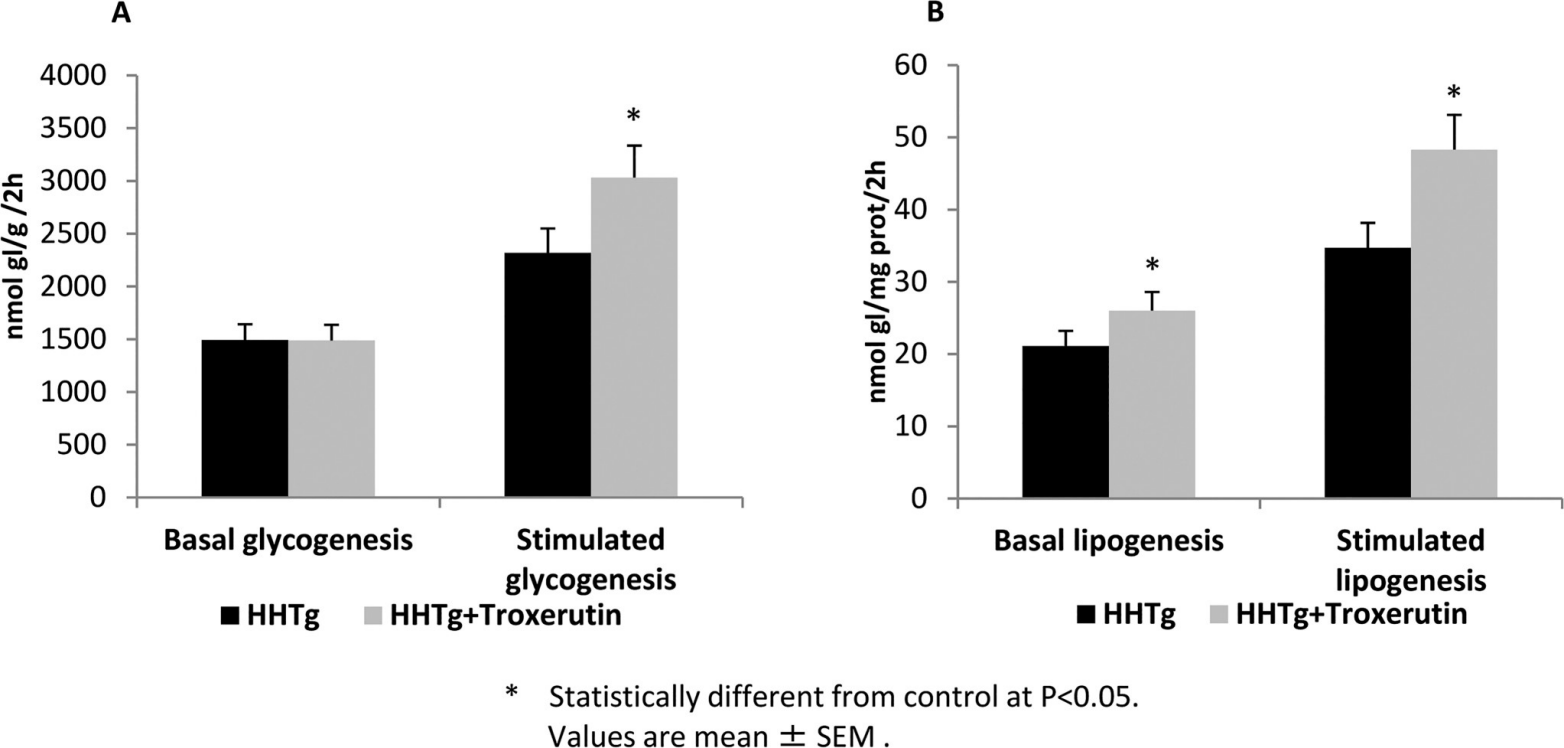
Basal and insulin-stimulated ^14^C-U glucose incorporation into lipids in epididymal adipose tissue (panel A) and into glycogen in muscle (panel B) in HHTg rats treated with troxerutin compared to controls. Data are measured in doublets using n = 8 rats per group per analyses. Data are expressed as means (SEM) and analysed by two-tailed unpaired Student’s t test. * p<0.05.

**Fig 2:**
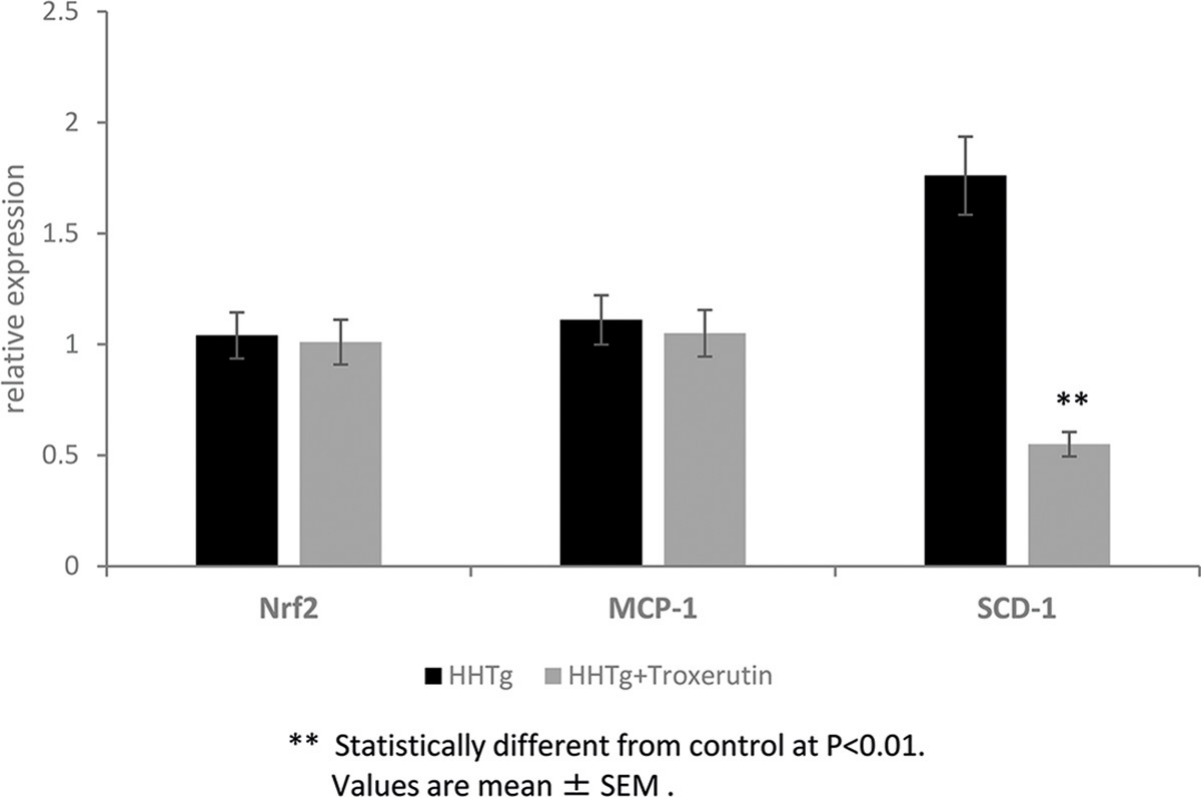
Hepatic expression of NRF2, MCP-1 and SCD-1 genes in HHTg rats treated with troxerutin compared to controls. Data are measured in triplets using n = 8 rats per group per analyses. Data are expressed as means (SEM) and analysed by two-tailed unpaired Student’s t test. ** p<0.01.

**Fig 3:**
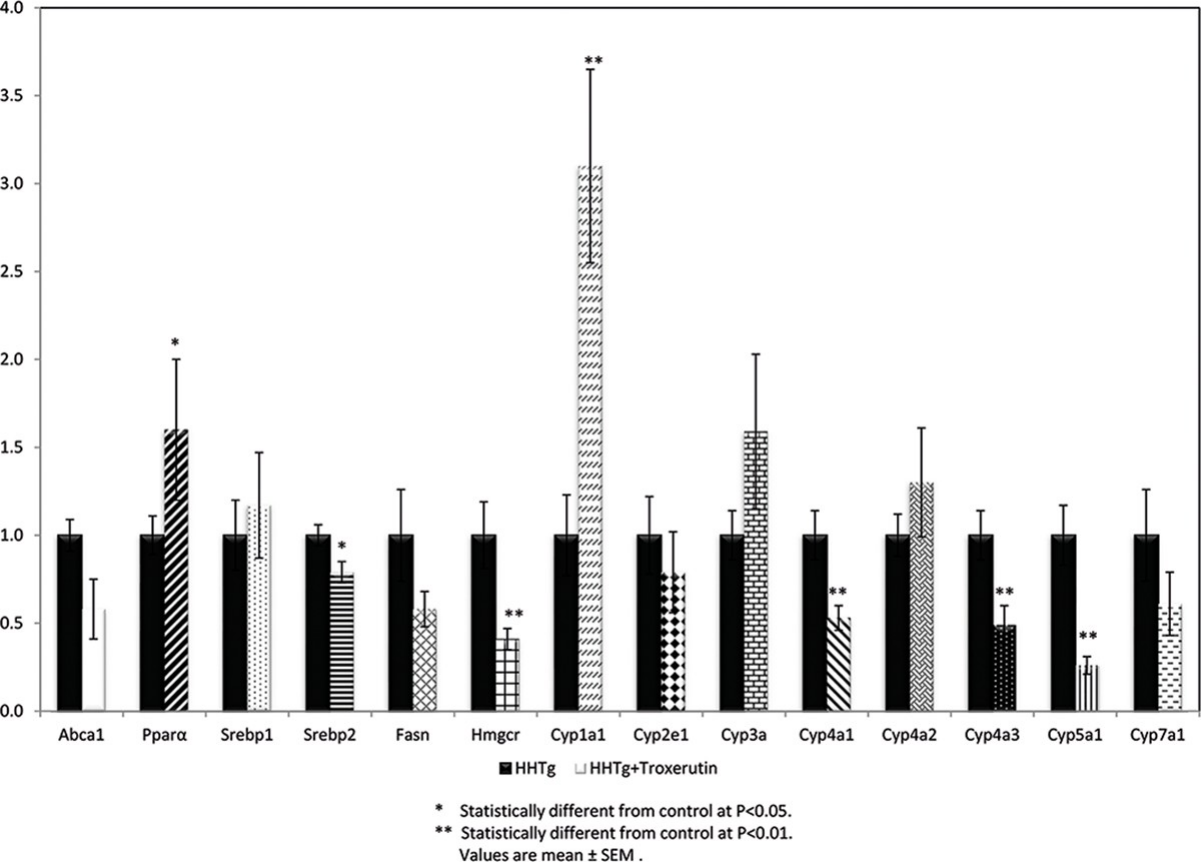
Hepatic gene expression of lipid metabolism and the cytochrome P450 system in HHTg rats treated with troxerutin compared to controls. Data are measured in triplets using n = 8 rats per group per analyses. Data are expressed as means (SEM) and analysed by two-tailed unpaired Student’s t test. * p<0.05, ** p<0.01.

TRX treatment did not affect food intake, body weight, or the weight of epididymal or retroperitoneal fat pads in HHTg rats. However, TRX significantly decreased non-fasting serum glucose and insulin levels in the same animals (Table 1). As shown in Fig 1, TRX-treated HHTg rats exhibited higher insulin-stimulated incorporation of ^14^C-U glucose into skeletal muscle glycogen. In visceral adipose tissue, basal and insulin-stimulated incorporation of ^14^C-U glucose into lipids were significantly increased in TRX-treated animals (Fig 1). Higher metabolic activity of visceral adipose tissue was demonstrated by significantly increased adrenaline-stimulated lipolysis in TRX-treated rats (2.31±0.09 vs 2.04±0.11 μmol/g; p<0.05). Blood lipids (triacylglycerols, total and HDL-cholesterol, NEFA) were unaffected by TRX, whereas cholesterol accumulation in the liver and ectopic triacylglycerol accumulation in skeletal muscles (*musculus soleus*) were decreased. The hypocholesterolaemic effect in the liver was associated with decreased relative expression of 3-hydroxy-3-methyl-glutaryl-coenzyme A reductase (HMGCR, a key enzyme involved in cholesterol synthesis) and stearoyl-CoA desaturase 1(SCD-1, a key lipogenic enzyme) (Figs 2 and 3). The decreased level of cholesterol after TRX administration was possibly related to reduced gene expression of the transcription factor SREBP2 (Fig 3), which controls cholesterol homeostasis by stimulating the transcription of sterol-regulated genes. In the liver, elevated gene expression of PPARα in TRX-treated rats (Fig 3) can increase lipid oxidation and thus it inhibits an excess of hepatic lipid accumulation.

**Table 1:** Metabolic parameters in HHTg rats supplemented with troxerutin.

	HHTg	HHTg+troxerutin
Body weight, g	414±4	413±7
Weight of epididymal fat pads/100 g bwt	1.84±0.05	1.77±0.08
**Serum**		
Non-fasting glucose, mmol/l	8.8±0.1	7.8±0.3*
Insulin, nmol/l	0.293±0.057	0.164±0.032*
HMW adiponectin, μg/ml	4.00±0.17	4.68±0.09*
Triacylglycerols, mmol/l	4.95±0.22	4.79±0.36
Total cholesterol, mmol/l	1.59±0.05	1.53±0.09
HDL-cholesterol, mmol/l	0.43±0.05	0.40±0.08
NEFA, mmol/l	0.52±0.03	0.47±0.03
MCP-1, ng/ml	3.36±0.71	2.88±0.26
IL-6, pg/ml	125±14	130±19
CRP, μg/ml	324±16	296±32
**Liver**		
Triacylglycerols, μmol/g	8.92±0.66	8.95±0.35
Total cholesterol, μmol/g	6.55±0.18	5.51±0.22**
**Musculus soleus**		
Triacylglycerols, μmol/g	4.10±0.56	2.70±0.29*

Values are mean ± SEM for each group, n = 8 for both group

* p<0.05

** p<0.01.

In addition, TRX supplementation had no significant effect on plasma concentrations of pro-inflammatory parameters CRP, MCP-1 or IL-6 (Table 1). But TRX-treated rats exhibited the increase in some of insulin sensitivity parameters–TRX treatment increased HMW plasma adiponectin concentrations (Table 1) and also increased the sensitivity of peripheral tissues to insulin action.

In HHTg rats, TRX supplementation was associated with an amelioration of oxidative stress, as indicated by the decreased concentration of intermediate products–conjugated dienes and end-product TBARS–in the liver (Table 2). Inhibition of oxidative stress may have been due to the improved antioxidant defence. Although GSH concentrations remained unchanged, GSSG levels significantly decreased. As a consequence, the GSH/GSSG ratio elevated, which implies that it may have positively influenced the activity of GSH-dependent antioxidant enzymes. As shown in Table 2, glutathione peroxidase was increased after TRX administration. Although the activity of glutathione reductase and glutathione transferase had a tendency to increase, the change was not significant compared to the control group. TRX activated liver superoxide dismutase, but the activity of catalase remained unchanged. In spite of the activated antioxidant enzymes, there was no increase in the expression of NRF2/NFE2L2 (nuclear factor-erythroid 2-related factor 2), a key transcription factor responsible for the constitutive and inducible expression of ARE-regulated genes in antioxidant enzymes (Fig 2).

**Table 2:** Oxidative stress parameters in the liver of HHTg rats supplemented with troxerutin.

	HHTg	HHTg+troxerutin
**Lipid peroxidation products**		
TBARS, nmol/mg protein	1.69±0.18	1.14±0.01*
Conjugated dienes, nmol/mg protein	31.5±2.4	23.0±1.5*
**Glutathione forms and ratio**		
GSH, μmol/ mg protein	42.2±2.0	42.3±6.2
GSSG, μmol/mg protein	3.89±0.19	2.80±0.18**
GSH/GSSG	11.2±1.0	15.0±1.2*
**Activity of antioxidant enzymes**		
Superoxide dismutase, U/mg protein	0.12±0.01	0.16±0.01*
Catalase, μmol H_2_O_2_/min/mg protein	1341±87	1466±80
Glutathione peroxidase, μmol NADPH/min/mg protein	307±13	373±22*
Glutathione reductase, nmol NADPH/min/mg protein	122±8	139±14

Values are mean ± SEM for each group, n = 8 for both group

* p<0.05

** p<0.01.

As shown in Fig 3, TRX-treated HHTg rats exhibited significant changes in some of cytochrome P450 family enzymes. Hepatic gene expression of CYP4A1, CYP4A3 and CYP5A1 were decreased significantly after TRX treatment compared to untreated rats, whereas a marked elevation in the relative gene expression of CYP1A1 in the liver was observed (Fig 3).

## Discussion

Along with the presence of hypertension and visceral obesity, insulin resistance and fatty liver steatosis are among the main factors associated with metabolic syndrome, and thus increase the risk of the onset of diabetes and cardiovascular complications. Several lines of evidence suggest that bioflavonoids such as TRX can have beneficial effects on these complications by improving glycaemic control, lipid profiles and antioxidant status.

In this study, we tested the effect of TRX on glucose and lipid metabolism, oxidative stress and inflammation using a non-obese model of metabolic syndrome. This model exhibits all disorders of this syndrome but without the complications of diet-induced obesity. Our results demonstrate that TRX significantly reduced hepatic cholesterol accumulation and ameliorated oxidative stress in the liver and insulin resistance in peripheral tissue.

Bioflavonoids like TRX possess expressive antioxidant properties that can suppress oxidative stress and exert anti-inflammatory effects–two important mechanisms in the development of liver steatosis. Many authors suggest that activation of antioxidant enzymes is a common hepatoprotective mechanism of flavonoid compounds, including TRX [18, 19]. Our study revealed increased activity of hepatic antioxidant enzymes and an elevated GSH/GSSG ratio in HHTg rats. Glutathione is a sensitive marker of hepatic oxidative damage, and its elevation in our study may be associated with increased hepatic glutathione peroxidase activity. In accordance with our results, other studies have reported increased hepatic glutathione levels in high-fat diet-fed mice when treated with TRX [12]. Increased activity of glutathione peroxidase and SOD may play an important role in decreasing lipid peroxidation by participating in the removal of lipoperoxidation products. These findings are consistent with our observations of lower concentrations of lipoperoxidation products in the livers of TRX-treated rats. Our results indicate that activation of antioxidant enzymes was not associated with the alteration of the transcription factor NRF2, a crucial regulator of the cellular defence against oxidative stress. The mechanism, by which TRX regulates oxidative stress, is direct inactivation of free radicals and the stimulation of antioxidant enzymes activity in particular SOD by the binding of TRX to the proteins. Increased SOD activity can abrogate mitochondrial oxidative stress that is believed one of the main source of intracellular radicals.

The possible anti-diabetic properties of flavonoid TRX have also been investigated. In the present study, TRX treatment reduced non-fasting blood glucose and hyperinsulinaemia. The beneficial effects of TRX on blood glucose and insulin have been documented in high-fat/high-fructose diet-fed mice [10, 20] and diet-induced, obese, diabetic rats [7]. Increasing glucose uptake of peripheral tissues and inhibiting hepatic gluconeogenesis can belong to possible mechanism by which TRX contributes to decreased blood glucose [12]. One of the most serious metabolic syndrome-associated complications affecting glucose utilisation is increased lipid deposition in non-adipose tissue. In HHTg rats, improved muscle insulin sensitivity was associated with decreased ectopic triacylglycerols accumulation in muscles. In a previous study, we showed that triacylglycerols content in skeletal muscles is significantly more elevated in HHTg rats than in Wistar control rats [21]. One of the possible mechanisms by which lipid content increases muscle-mediated insulin resistance is via the generation of lipotoxic intermediates, primarily diacylglycerols and ceramides, which interfere with insulin signalling [22].

Increased adiponectin levels may also contribute to improving insulin sensitivity in TRX-treated HHTg rats. Another possible mechanism involved in the anti-diabetic effect of TRX is increased phosphorylation of IRS-1 and AKT. As observed in the muscles of diabetic rats, this mechanism restores the level of intracellular glucose transporter GLUT4, thereby improving glucose uptake, oxidation and glycogen content [7, 23]. In our study, TRX treatment is also associated with higher basal and insulin-stimulated incorporation of glucose into adipose tissue lipids. TRX did not significantly affect any of the known parameters that influence the use of glucose in adipose tissue, such as elevated NEFA, the re-esterification of fatty acids, adipose tissue protein content, or inflammation. However, serum levels of NEFA, CRP or MCP-1 tended to be decreased. Furthermore, increased adrenaline-stimulated lipolysis together with increased adiponectin secretion in HHTg rats after TRX treatment can associated with higher metabolic activity of visceral adipose tissue and can contribute to higher glucose uptake. In summary, TRX may improve insulin sensitivity by improving insulin-signalling molecules, boosting anti-inflammatory effects, and assisting adiponectin secretion and glucose utilisation.

The development of fatty liver as an important feature of metabolic syndrome is associated with impaired lipid metabolism and hepatic lipid accumulation. TRX has attracted attention because of its reported possible effects on lipid homeostasis. In the presented study, TRX reduced hepatic cholesterol accumulation although serum cholesterol levels were not changed. To search for the mechanisms in the cholesterol-lowering effect of TRX in HHTg rats, we analysed gene expression profiles in the liver. Our analysis reveals the likelihood that hepatic genes involved in cholesterol synthesis and oxidation play a role in the mechanism of TRX action. As our results show, TRX caused decreased in HMGCR and increased in PPARα gene expression. The transcription factor PPARα regulates the genes involved in both fatty acid uptake and metabolism, increases lipid oxidation, and inhibits excess hepatic lipid accumulation. TRX exerts a lipid-lowering effect via upregulation of PPARα following induced expression of the genes involved in fatty acid oxidation, and via downregulation of SREBP-1c expression, resulting in reduced fatty acid and triacylglycerol synthesis [7].

The lipid-lowering effect of TRX may be partly attributable to inhibited expression of SCD1 –a key lipogenic enzyme. Increased expression of SCD1 is associated with the development of dyslipidaemia, atherosclerosis and diabetes [24]. Induction of lipogenic genes such as SCD1 may enhance hepatic steatosis. In our study, TRX treatment led to a significant decrease in SCD1 gene expression, which suggests that it probably affects lipid metabolism via various mechanisms. It is possible that decreases in lipogenic enzymes such as SCD1 may also be associated with increased CYP1A1 activity. In our study, the increase in CYP1A1 mRNA expression was observed following TRX administration. Indeed, a similar effect on CYP1A1 has also been observed in other studies dealing with bioflavonoids [25, 26].

Recent results obtained using CYP1A1(-/-) mice demonstrate that elevated expression of CYP1A1 protects against the development of non-alcoholic fatty liver disease [27], and dysregulation of the CYP1A1 gene is reported to be involved in the transport and metabolism of lipids [28].

Other CYP450 family enzymes may contribute to the beneficial effects of TRX. The expression and activity of hepatic CYP isoforms may be dysregulated in diabetic and insulin-resistant states. As previously described, in studies with ZDF [29] and Goto-Kakizaki rats [30], the CYP4A isoform is upregulated in pathological conditions such as diabetes. CYP4A isoforms–hydroxylate epoxyeicosatrienoic acids (EETs) and hydroxyl-epoxyeicosatrienoic acids (HEETs)–act as PPARα agonists [31]. Therefore, the decreased CYP4A1 mRNA expression observed in our study may assist in improving lipid metabolism and could be possibly involved in the metabolism of eicosanoids and affect their pro-inflammatory properties, which are formed by CYP4A isoforms that catalyse metabolites of arachidonic acid.

TRX treatment was also associated with reduced CYP5A1 mRNA expression. This CYP450 family isoform metabolises the cyclooxygenase product prostaglandin H2 (PGH2) into thromboxane A2, a potent inducer of vasoconstriction and platelet aggregation. Through decreased CYP5A1, TRX administration can improve thrombotic status and vascular complications. Furthermore, the lipid composition that modulates the activity of endoplasmic reticulum-bound protein CYP5A1 is important for increasing thromboxane formation [32]. Taken together, TRX may improve hepatic cholesterol level by several mechanisms–affecting gene expression of enzymes and transcription factors involved in cholesterol metabolism and also altering in some of CYP450 family enzymes.

To the best of our knowledge, this is the first study to investigate the effect of TRX on pre-existing disorders using a non-obese model of metabolic syndrome. Although about 20% of patients with metabolic syndrome are not obese, obesity nevertheless plays an important role in the pathogenesis of metabolic syndrome [33]. However, since no clinical studies on TRX treatment have been carried out to date, our results would benefit from being supported by a well-designed clinical study of prediabetic patients.

## Conclusion

Our results indicate that TRX supplementation can ameliorate metabolic disturbances in non-obese model of metabolic syndrome. Besides the antioxidant properties, TRX improved insulin sensitivity in peripheral tissues and alleviated hepatic cholesterol accumulation. Alterations of some CYP enzymes activity and genes involved in cholesterol metabolism can be involved in the mechanism. Thus TRX could appropriate the treatment of metabolic syndrome. However, for human application, these results need to be verified by clinical studies.

## Supporting information

S1 File Arrive guidelines checklist. (DOCX)

## Abbreviations

CYP: cytochrome P450
NEFA: non-esterified fatty acids
GLUT: glucose transporter
GSH: reduced form of glutathione
GSSG: oxidized form of glutathione
IL-6: interleukin 6
MCP-1: monocyte chemoattractant protein-1
PPAR: peroxisome proliferator-activated receptor
SCD: stearoyl-CoA desaturase
SREBP: sterol regulatory element-binding protein
TBARS: thiobarbituric acid reactive substance
TRX: troxerutin
