# Supplementary material for: Beneficial effects of troxerutin on metabolic disorders in non-obese model of metabolic syndrome
Source: PLoS One. 2019 Aug 12;14(8):e0220377. doi: 10.1371/journal.pone.0220377 (PMC6690532; doi:10.1371/journal.pone.0220377)

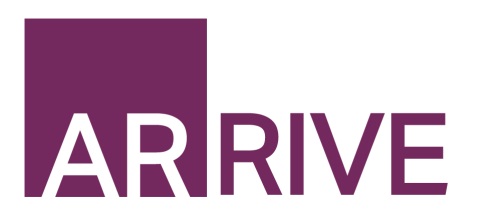


The ARRIVE Guidelines Checklist

Animal Research: Reporting In Vivo Experiments

Carol Kilkenny^1^, William J Browne^2^, Innes C Cuthill^3^, Michael Emerson^4^ and Douglas G Altman^5^

*^1^The National Centre for the Replacement, Refinement and Reduction of Animals in Research, London, UK, ^2^School of Veterinary Science, University of Bristol, Bristol, UK, ^3^School of Biological Sciences, University of Bristol, Bristol, UK, ^4^National Heart and Lung Institute, Imperial College London, UK, ^5^Centre for Statistics in Medicine, University of Oxford, Oxford, UK.*

|  | | ITEM | RECOMMENDATION | Section/ Paragraph |
| --- | --- | --- | --- | --- |
| 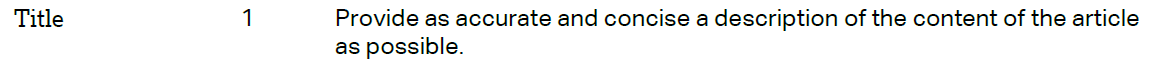 | | | Page 1 |  |
| 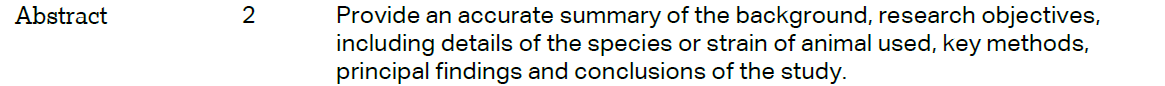 | | | Page 2-3 |  |
| INTRODUCTION | | |  |  |
| 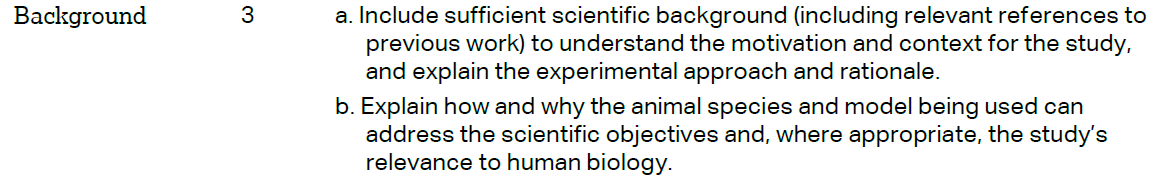 | | | Page 3-5 |  |
| 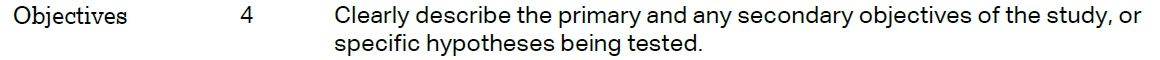 | | | Page 4 |  |
| METHODS | | |  |  |
| 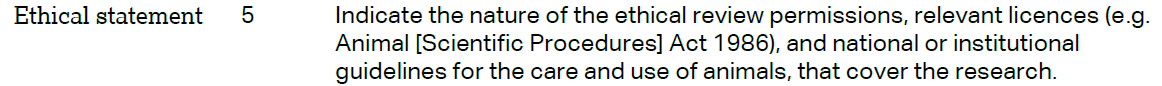 | | | Page 5 |  |
| 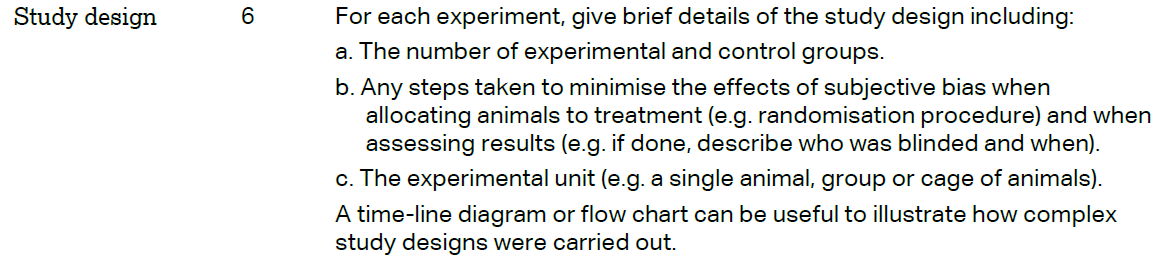 | | | Page 5 |  |
| 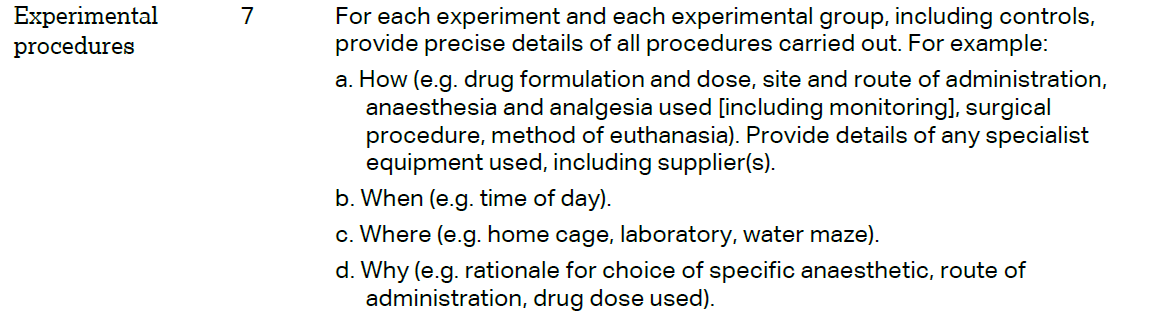 | | | Page 5 |  |
| 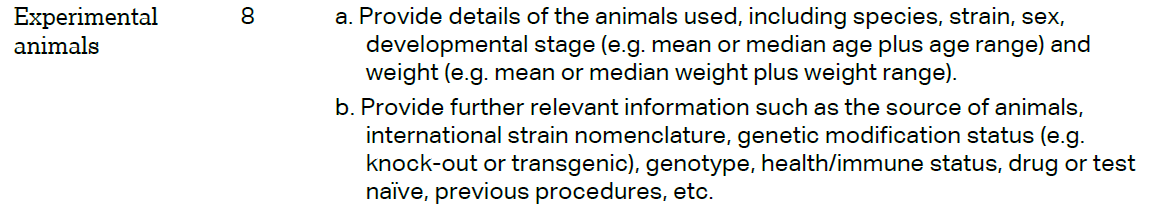 | | | Page 5 |  |

The ARRIVE guidelines. Originally published in *PLoS Biology*, June 2010^1^

| 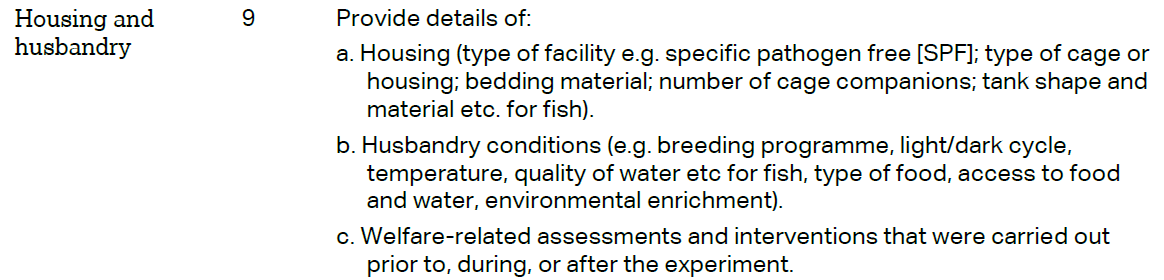 | Page 5 | |
| --- | --- | --- |
| 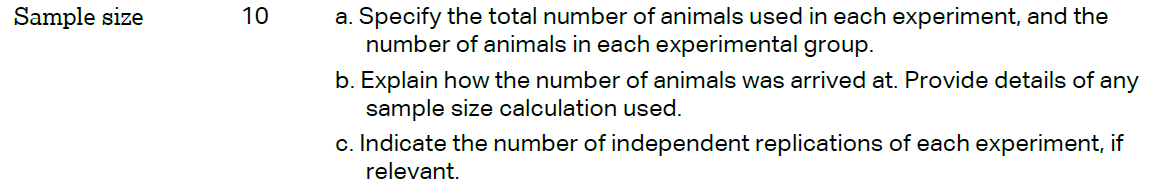 | Page 5  Table 1-2 | |
| 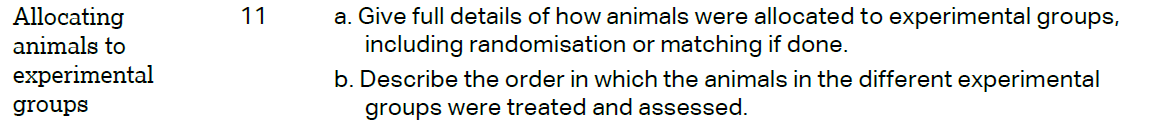 | Page 5 | |
| 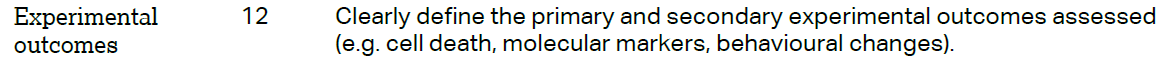 | Page 5-6 | |
| 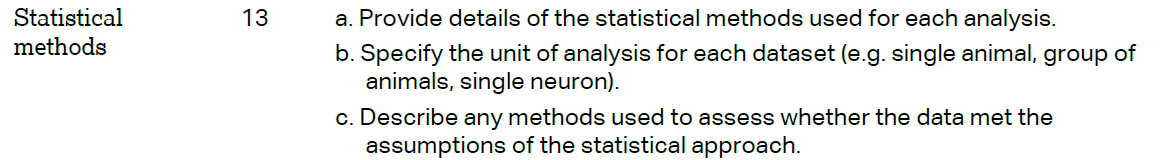 | Page 7 | |
| RESULTS |  | |
| 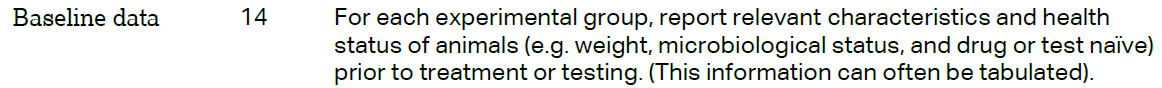 | Table 1 | |
| 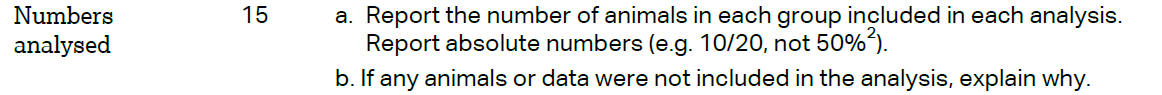 | Table 1-2 | |
| 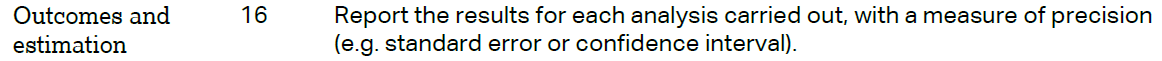 | Results section  Table 1-2 | |
| 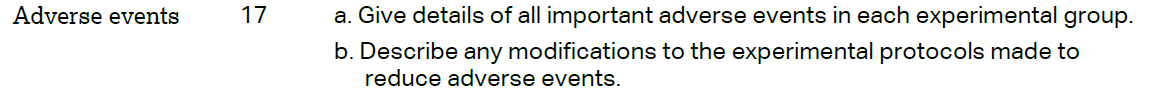 | Results section | |
| DISCUSSION |  | |
| 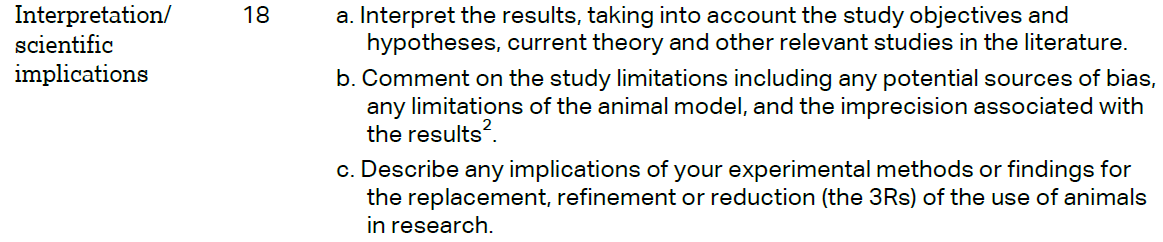 | Discussion section | |
| 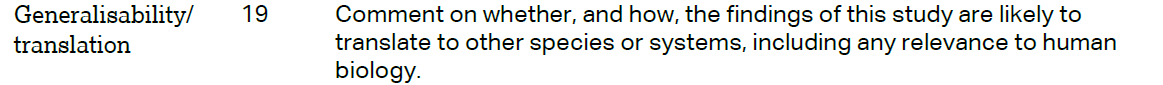 | Page 12-13 | |
| 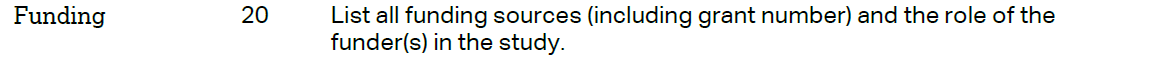 | | Page 13 |


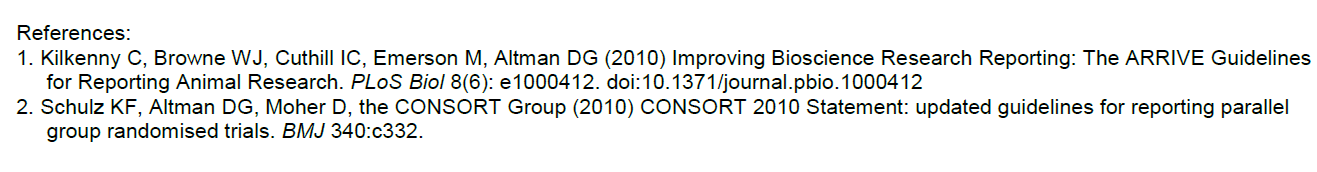

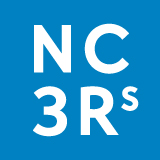

Supplement: S1 File — (DOCX) [file pone.0220377.s001.docx]
